# Synergistic Effects of Cilostazol and Probucol on ER Stress-Induced Hepatic Steatosis via Heme Oxygenase-1-Dependent Activation of Mitochondrial Biogenesis

**DOI:** 10.1155/2016/3949813

**Published:** 2016-01-06

**Authors:** Yingqing Chen, Indira Pandiri, Yeonsoo Joe, Hyo Jeong Kim, Seul-Ki Kim, Jeongmin Park, Jinhyun Ryu, Gyeong Jae Cho, Jeong Woo Park, Stefan W. Ryter, Hun Taeg Chung

**Affiliations:** ^1^Department of Biological Sciences, University of Ulsan, Ulsan 680-749, Republic of Korea; ^2^Department of Anatomy, School of Medicine and Institute of Health Sciences, Gyeongsang National University, Jinju 660-701, Republic of Korea; ^3^Joan and Sanford I. Weill Department of Medicine, New York-Presbyterian Hospital, and Division of Pulmonary and Critical Care Medicine, Weill Cornell Medical Center, New York, NY 10065, USA

## Abstract

The selective type-3 phosphodiesterase inhibitor cilostazol and the antihyperlipidemic agent probucol have antioxidative, anti-inflammatory, and antiatherogenic properties. Moreover, cilostazol and probucol can regulate mitochondrial biogenesis. However, the combinatorial effect of cilostazol and probucol on mitochondrial biogenesis remains unknown. Endoplasmic reticulum (ER) stress is a well-known causative factor of nonalcoholic fatty liver disease (NAFLD) which can impair mitochondrial function in hepatocytes. Here, we investigated the synergistic effects of cilostazol and probucol on mitochondrial biogenesis and ER stress-induced hepatic steatosis. A synergistic effect of cilostazol and probucol on HO-1 and mitochondrial biogenesis gene expression was found in human hepatocellular carcinoma cells (HepG2) and murine primary hepatocytes. Furthermore, in an animal model of ER stress involving tunicamycin, combinatorial treatment with cilostazol and probucol significantly increased the expression of HO-1 and mitochondrial biogenesis-related genes and proteins, whereas it downregulated serum ALT, eIF2 phosphorylation, and CHOP expression, as well as the lipogenesis-related genes SREBP-1c and FAS. Based on these results, we conclude that cilostazol and probucol exhibit a synergistic effect on the activation of mitochondrial biogenesis* via *upregulation of HO-1, which confers protection against ER stress-induced hepatic steatosis.

## 1. Introduction

Mitochondria are ubiquitous membrane-bound organelles essential for cellular energy generation, which contribute many important metabolic functions including pyruvate and fatty acid oxidation, nitrogen metabolism, and heme biosynthesis. The mitochondria are the site of the electron transport chain which provides the bulk of cellular energy in the form of ATP [1, 2]. Mitochondrial biogenesis refers to the process by which new mitochondria are formed in the cell. The peroxisome proliferator-activated receptor-gamma (PPAR*γ*) family of transcriptional coactivators includes PPAR*γ*-coactivator (PGC)-1 alpha (PGC-1*α*), PGC-1*β*, and the PGC-related coactivator, PRC. Of these, PGC-1*α* has been identified as a master regulator of mitochondrial biogenesis. PGC-1*α* can coactivate the nuclear respiratory factor-1 (NRF-1) and subsequently the mitochondrial transcription factor A (TFAM), which is directly responsible for transcribing nuclear-encoded mitochondrial proteins [3–5]. Hepatocytes are rich in mitochondria, which play an important role in hepatocyte metabolism. Fatty acid oxidation mainly occurs in the liver for energy production. Impaired mitochondrial *β*-oxidation may cause NAFLD [6]. Expression of mtDNA-encoded polypeptides [7] and activity of complexes I, III, IV, and V [8] were reduced in patients with NASH.

Heme oxygenase-1 (HO-1) is a major stress-inducible protein in mammalian cells. In previous studies, HO-1 has been shown to regulate mitochondrial biogenesis in cardiomyocytes* via* NF-E2-related factor-2- (Nrf2-) mediated transcriptional control of nuclear respiratory factor-1 (NRF-1) [9]. The promoter region of the HO-1 gene contains multiple copies of antioxidant response elements that are critical for stress-inducible gene expression and that are tightly regulated by the transcription factor Nrf2 [10]. Recently, we demonstrated that cilostazol increases the expression of genes involved in mitochondrial biogenesis, including NRF-1, PGC-1*α*, and TFAM* via* upregulating the production and activity of HO-1 in a human hepatoma cell line (HepG2) [11]. Cilostazol (6-[4-(1-cyclohexyl-1H-tetrazol-5-yl)butoxy]-3,4-dihydro-2(1H)-quinolinone) has been demonstrated as a selective inhibitor of type-3 phosphodiesterase (PDE3), which can increase the intracellular level of 3′-5′-cyclic adenosine monophosphate (cAMP) [12]. As an antithrombotic drug, cilostazol is widely used for the treatment of thrombotic vascular disease due to its antiplatelet aggregation properties [13]. Moreover, cilostazol inhibits LPS-induced apoptosis* via* reducing the production of intracellular reactive oxygen species (ROS) [14] and protects mice against endotoxin shock* via* MAPK inhibition and NF-*κ*B inactivation [15]. Cilostazol can also protect mice against carbon tetrachloride-induced liver fibrosis by attenuating hepatic stellate cell activation [16]. In addition, cilostazol has been shown to promote mitochondrial biogenesis in human umbilical vein endothelial cells (HUVECs) through activating the expression of PGC-1*α* [17].

Probucol (4,4′-[propane-2,2-diylbis(thio)]bis(2,6-di-tert-butylphenol)) is a potent lipid-soluble antioxidant, which has been reported to possess strong antiatherogenic properties [18]. Additionally, numerous studies have described anti-inflammatory effects of probucol, including inhibition of adhesion of mononuclear cells to the vascular endothelium in cholesterol-fed rabbits and downregulation of vascular cell adhesion molecule-1 (VCAM-1) expression [19, 20]. A previous study showed that probucol induces the expression and activity of HO-1, which contributes to the inhibition of vascular smooth muscle cell proliferation for therapeutic intervention against occlusive vascular disease [21]. Probucol also increases HO-1 expression and activity in balloon-injured rabbit aortas and rabbit aortic smooth muscle cells to protect against atherosclerosis [22].

Based on the pharmacological profiles of cilostazol and probucol, the combination of these two drugs has shown synergistic effects on reducing ischemic infarct in the rat brain compared to cilostazol or probucol monotherapy [23]. In low density lipoprotein receptor-deficient mice fed with a high fat diet, the combinatorial effects of cilostazol and probucol significantly decreased atherosclerotic lesions relative to that of cilostazol and probucol alone [24]. The combinatorial effects of cilostazol and probucol also attenuated hypercholesterolemia-induced exacerbation in ischemic brain injury* via* decreasing MCP-1 expression and CD11b and GFAP immune reactivity in the ischemic cortex from apolipoprotein E (ApoE) knockout mice [25].

Because cilostazol and probucol both increase HO-1 expression and have antioxidant properties, we hypothesized that the combination of low doses of these two drugs may exert synergistic effects on mitochondrial biogenesis* via* increasing the production and activity of HO-1 in a HepG2 human hepatoma cell line. Our results demonstrate that the combination of cilostazol and probucol significantly increased the expression of HO-1, PGC-1*α*, NRF-1, and TFAM relative to cilostazol and probucol individually. Furthermore, we also demonstrated synergistic effects of cilostazol and probucol on increasing ATP production and mitochondrial DNA (mtDNA) content.

## 2. Materials and Methods

### 2.1. Reagents

Cilostazol (OPC-13013), [6-[4-(1-cyclohexyl-1H-tetrazol-5-yl)butoxy]-3,4-dihydro-2(1H)-quinolinone], and probucol, [4,4′-(isopropylidenedithio)  bis(2,6-di-tert-butylphenol)], were donated by Otsuka Pharmaceutical Co. Ltd. (Tokushima, Japan). Tunicamycin (TM) was purchased from Sigma-Aldrich (St Louis, MO, USA).

### 2.2. Cell Culture

HepG2 cells were obtained from the Korean cell line bank (Seoul, Korea). HepG2 cells were cultured in DMEM (Gibco, Grand Island, NY) containing 10% FBS and 1% penicillin-streptomycin solution at 37° in humidified incubators containing 5% CO_2_. HepG2 cells were treated with cilostazol at various concentrations (0, 0.1, 0.3, 1, and 3 *μ*M) with or without probucol (0.1 *μ*M) for 4 h.

### 2.3. Animals

Animals were maintained in a specific pathogen-free facility. Animal studies were approved by the university of Ulsan Animal Care and Use Committee. The mice were maintained under specific pathogen-free conditions at 18–24° and 40–70% humidity, with a 12-hour light-dark cycle. Food and drinking water were available* ad libitum*. Male C57BL/6 wild-type mice (6 weeks old) were purchased from ORIENT (Pusan, Korea). C57BL/6 mice were assigned randomly into five groups (CON, TM, TM + PB, TM + CZ, and TM + PB + CZ), with six mice in each group. C57BL/6 mice were injected with cilostazol (3 mg/kg body weight) and probucol (1 mg/kg body weight) individually or combinatorially once daily for 3 days and then they were sacrificed after challenging with tunicamycin (3 mg/kg body weight) for 24 h. BALB/c HO-1 knockout mice were provided by Dr. Mark A. Perrella (Brigham and Women's Hospital, Boston, MA).

### 2.4. Isolation of Primary Hepatocytes

Primary hepatocytes from wild-type (WT) and HO-1 knockout (KO) mice were isolated. Livers were perfused with Ca^2+^- and Mg^2+^-free HBSS containing EGTA (2.5 mM) and then digested with a collagenase buffer containing collagenase (0.5 mg/mL, C5138, Sigma), NaCl (66.7 mM), KCl (6.7 mM), HEPES (50 mM), and CaCl_2_ (4.8 mM). Digested livers were dissected and then gently teased with forceps until they were in solution. The cell suspensions were filtered through a 100 *μ*m nylon cell strainer (BD Biosciences). The cells were centrifuged for 3 min at 700 rpm and resuspended with HBSS. After the pellet suspensions were centrifuged with 25% Percoll for 5 min at 800 rpm with the brake option off, the pellets were washed with DMEM supplemented with 10% FBS, and then cells were seeded into collagen precoated 100 mm tissue culture plates. After 24 h, nonadherent cells were removed by aspiration, and fresh medium was added.

### 2.5. Western Blot Analyses

Harvested liver tissues and cells were lysed with mammalian lysis buffer containing phosphatase and protease inhibitors. Equal amounts of cell lysates were measured with the BCA protein assay reagent (Pierce Biotechnology, Rockford, IL). Lysates were boiled in sample buffer containing *β*-mercaptoethanol for 5 min. Proteins were then subjected to SDS-PAGE and transferred to polyvinylidene difluoride membranes (GE healthcare). After blocking with 5% skim milk in PBS, membranes were incubated with appropriate dilutions of antibodies at 4°C overnight as follows: HO-1 (Enzo, ADI-OSA-150, 1 : 1000 dilution), PGC-1*α* (Abcam, ab72230, 1 : 1000 dilution), COX III (Abcam, ab110252, 1 : 20000 dilution), COX IV (Cell Signaling, #4844S, 1 : 1000 dilution), p-eIF2*α* (Cell Signaling, #9721S, 1 : 1000 dilution), and *β*-actin (Cell Signaling, #4967S, 1 : 2000 dilution). Membranes were then washed with 0.05% PBS-Tween 20 and incubated with a 1/5000 dilution of HRP-conjugated secondary Abs at room temperature for 1 h. Immunoreactivity was detected by using the ECL detection system (GE Healthcare). Films were exposed at multiple time points to ensure that the images were not saturated. The relative band density was analyzed by using ImageJ software (US National Institutes of Health, Bethesda, MD).

### 2.6. Reverse Transcriptase PCR

Total RNA from cells was isolated with TRIzol reagent (Invitrogen, Carlsbad, CA), according to the manufacturer's instructions. In brief, total RNA (2 *μ*g) was used to synthesize the first strand cDNA by using Oligo-dT primers (Bioneer, Daejeon, Korea) and M-MLV reverse transcriptase (Promega, Madison, WI). The synthesized cDNA was subjected to the PCR-based amplification. The following primers were used: hHO-1 forward, 5′-CAGGAGCTGCTGACCCTAGA-3′, reverse, 5′-AGCAACTGTCGCCACCAGAA-3′, hPGC-1*α* forward, 5′-TGAGAGGGCCAAGCAAAG, reverse, 5′-ATAAATCACGCGCTCTT-3′, hNRF-1 forward, 5′-CCATCTG GTGGCCTGAAG-3′, reverse, 5′-GTGCCTGGGTCCATGAAA-3′, hTFAM forward, 5′-GAACAACTACCCATATTTAAAGCTCA-3′, reverse, 5′-GAATCAGGAAGTTCCCTCCA-3′, hGAPDH forward, 5′-CAATGACCCCTTCATTGACCTC-3′, reverse, 5′-AGCATCGCCCCAC TTGATT-3′, mHO-1 forward, 5′-TCCCAGACACCGCTCCTCCAG-3′, reverse, 5′-GGATTTGGGGCTGCTGGTTTC-3′, mPGC1*α* forward, 5′-GGAACTGCAGGCCTAACTCC-3′, reverse, 5′-TTGGAGCTGTTTTCTGGTGC-3′, mNRF-1 forward, 5′-CTCCAAACCCAACCCTGTCT-3′, reverse, 5′-TGGTGGCCTGAGTTTGTGTT-3′, mTFAM forward, 5′-CAGCCAGGTCCAGCTCACTA-3′, reverse, 5′-ATTAGGAGGGTCTCGCTCCA-3′, and mGAPDH forward, 5′-AGGCCGGTGCTGCTCAGTATGTC-3′, reverse, 5′-TGCCTGCTTCACCACCTTCT-3′. The expression of GAPDH was measured as internal control. The relative band density was analyzed by using ImageJ software (US National Institutes of Health, Bethesda, MD).

### 2.7. Real-Time Quantitative RT-PCR

Total RNA was prepared using TRIzol reagent (Invitrogen). 2 *μ*g of total RNA was used to synthesize the first strand cDNA by using Oligo-dT primers and MMLV reverse transcriptase (Promega, Madison, WI, USA) according to the manufacturer's instructions. Real-time quantitative PCR was performed with SYBR Green qPCR Master Mix (2x, USB Production; Affymetrix) on an ABI 7500 Fast Real-Time PCR System (Applied Biosystems, Carlsbad, CA). Real-time PCR primer pairs were as follows: hHO-1 forward, 5′-CAGGAGCTGCTGACCCATGA-3′, reverse, 5′-AGCAACTGTCGCCA CCAGAA-3′, hPGC-1 forward, 5′-TGAGAGGGCCAAGCAAAG-3′, reverse, 5′-ATAAATCACACGGCGCTCTT-3′, hNRF-1 forward, 5′-CCATCTGGTGGCCTGAAG-3′, reverse, 5′-GTGCCTGGGTCCATGAAA-3′, hTFAM forward, 5′-GAACAACTACCCATATTTAAAGCT CA-3′, reverse, 5′-GAATCAGGAAGTTCCCTCCA-3′, hGAPDH forward, 5′-CAATGACCCCTTCATCCTC-3′, reverse, 5′-AGCATCGCCCCACTTGATT-3′, mHO-1 forward, 5′-TCAGTCCCAAACGTCGCGGT-3′, reverse, 5′-GCTGTGCAGGT GTTGAGCC-3′, mPGC-1 forward, 5′-AGCCGTGACCACTGACAACGAG-3′, reverse, 5′-GCTGCATGGTTCTGAGTGCTAAG-3′, mNRF-1 forward, 5′-CGCAGCACCTTTGGAGAA-3′, reverse, 5′-CCCGACCTGTGGAATACTTG-3′, mTFAM forward, 5′-GGAATGTGGAGCGTGCTAAAA-3′, reverse, 5′-T GCTGGAAAAACACTTCGGAATA-3′, mSREBP1 forward, 5′-TCCAGTGGCAAAGGAGGCA-3′, reverse, 5′-ATAGCAGGATGCCAACAGCA-3′, and mFAS forward, 5′-CGGAAACTTCAGGAAATGTCC-3′, reverse, 5′-TCAGAGACGTGTCACTCCTGG-3′.

### 2.8. Transfection of siRNAs

Small interfering RNAs (siRNAs) against human HO-1 (siHO-1) (SI02780533) were purchased from QIAGEN (Hilden, Germany) and negative control siRNA (scRNA) (AM4611) was purchased from Ambion (Austin, TX). HepG2 cells (7 × 10^5^) were transfected with siHO-1 and scRNA by using Lipofectamine 2000 (Invitrogen, Carlsbad, CA) for 24 h.

### 2.9. Hepatocellular Damage Assay

Hepatic injury was assessed by serum alanine transaminase (ALT) levels with use of the EnzyChrom Alanine Transaminase Assay Kit (BioAssay Systems, Hayward, CA).

### 2.10. ATP Measurements

Cellular ATP content was determined by means of the CellTiter-Glo Luminescent Cell Viability Assay (Promega, Madison, WI, USA) according to the manufacturer's instructions.

### 2.11. mtDNA Analysis

Total DNA was extracted from primary hepatocytes by use of AccuPrep Genomic DNA Extraction Kit (Bioneer, Daejeon, Korea). mtDNA copy number was measured by real-time quantitative PCR. The following primers for mtDNA were used: mouse cytochrome b (*Mus musculus domesticus* mitochondrion) forward primer, 5′-CCACTTCATCTTACCATTTA-3′, reverse primer, 5′-ATCTGCATCTGAGTTTAATC-3′. For nuclear DNA (nDNA) the following were used: mouse 18S rRNA forward primer, 5′-GGGAGCCTGAGAAACGGC-3′, reverse primer, 5′-GGGTCGGGAGTGGGTAATTT-3′. Relative amounts of mtDNA and nDNA copy numbers were compared.

### 2.12. Measurements of Triglyceride

Hepatic triglyceride was assessed by triglyceride colorimetric assay kit (Cayman Chemical, Ann Arbor, Michigan, USA). Briefly, liver tissues (50 mg), HepG2 cells (1 × 10^7^), and primary hepatocytes (1 × 10^7^) were homogenized in 200 *μ*L diluted Standard Diluents. After 10,000 ×g centrifugation for 10 minutes, supernatant were transferred to another tube. Before assaying, tissue samples required dilution at least 1 : 5, while there was no need for the dilution of serum or cell samples. Then we need 10 *μ*L serum and cell supernatant for the assay.

### 2.13. Measurements of Hepatic Triglyceride

Hepatic triglyceride was assessed using the triglyceride colorimetric assay kit (Cayman Chemical, Ann Arbor, Michigan, USA). Briefly, 50 mg liver tissues were homogenized in 200 *μ*L diluted Standard Diluents. After 10,000 ×g centrifugation for 10 minutes, supernatants were transferred to another tube. Before assaying, tissue samples required dilutions of at least 1 : 5 (v/v) by Standard Diluent.

### 2.14. Liver Histology

To detect the pathological changes, liver tissues were fixed in 10% neutral-buffered formalin solution and then dehydrated in graded alcohol, embedded in paraffin, sectioned into 4 *μ*m thick sections, and stained with hematoxylin and eosin (H&E).

### 2.15. Statistical Analysis

All values are expressed as means ± SE. Statistical differences between groups were evaluated by one-way ANOVA with* post hoc* Tukey's honestly significant difference (HSD) test. Data were analyzed and presented with GraphPad Prism software version 5 (GraphPad Software, San Diego, CA).

## 3. Results

### 3.1. Synergistic Effect of Cilostazol and Probucol on HO-1 Expression in HepG2 Cells and Primary Hepatocytes

**Figure 1:**
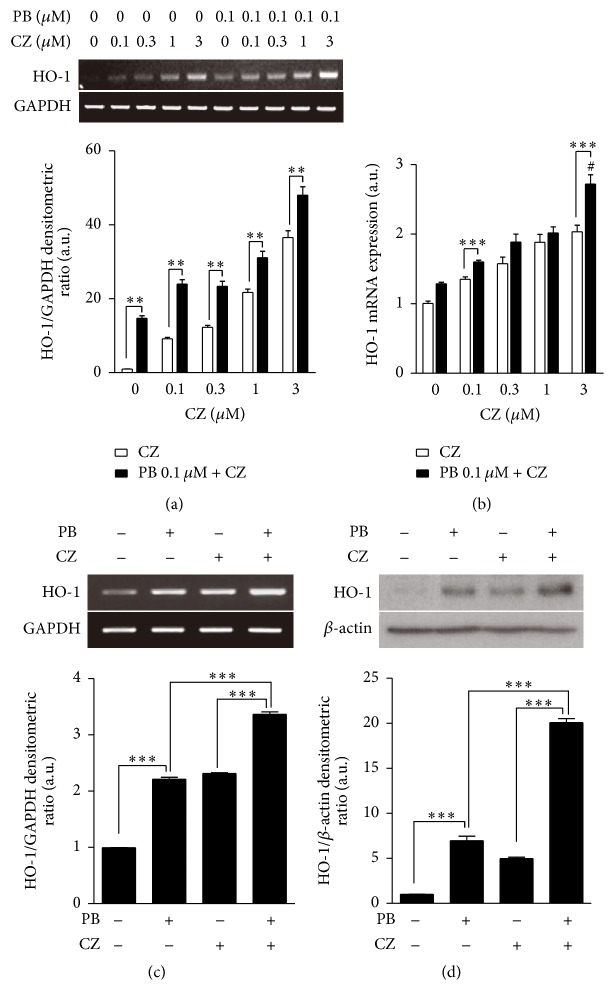
Effect of combinatorial treatment of cilostazol (CZ) and probucol (PB) on HO-1 expression. HepG2 cells were treated with cilostazol at various concentrations (0, 0.1, 0.3, 1, and 3 *μ*M) with or without probucol (0.1 *μ*M) for 4 h and HO-1 mRNA levels were detected by RT-PCR (a) and real-time RT-PCR (b). Primary hepatocytes were treated with probucol (0.1 *μ*M) and cilostazol (3 *μ*M) individually as well as with their combination for 4 h and HO-1 mRNA and protein levels were detected by RT-PCR (c) and Western blot analysis (d), respectively. Bar graphs, lower panels of (a), (c), and (d), are summary data of normalized densitometric ratios. Quantitative data are expressed as means ± SE; *n* = 3. ^*∗∗*^ *P* < 0.01; ^*∗∗∗*^ *P* < 0.001; #: probucol (0.1 *μ*M) and cilostazol (3 *μ*M) have a synergistic effect on HO-1 expression.

Due to previous studies reporting that both cilostazol and probucol can enhance HO-1 expression, we first evaluated the effect of the combined treatment of cilostazol and probucol on HO-1 mRNA expression by RT-PCR and real-time PCR. We treated HepG2 cells with cilostazol at various concentrations (0, 0.1, 0.3, 1, and 3 *μ*M) with or without probucol (0.1 *μ*M) for 4 h. As shown in Figures 1(a) and 1(b), cilostazol enhanced the mRNA expression of HO-1 in a dose-dependent manner, whereas the combined treatment with cilostazol and probucol increased HO-1 mRNA expression much higher than the corresponding dose of cilostazol alone. To confirm the synergistic effect of cilostazol and probucol on HO-1 expression, we treated primary hepatocytes from C57BL/6 mice with probucol (0.1 *μ*M) and cilostazol (3 *μ*M) individually or in combination for 4 h. Then we assessed the expression of HO-1 mRNA (Figure 1(c)) and protein levels (Figure 1(d)). Consistent with results from HepG2 cells, the combination treatment induced a significant increase in HO-1 expression compared to the individual treatments alone. These results suggest that cilostazol and probucol can exert a synergistic effect on the upregulation of HO-1 mRNA expression in HepG2 cells and in primary hepatocytes.

### 3.2. Combination of Cilostazol and Probucol Exerts Beneficial Effects on Mitochondrial Biogenesis

**Figure 2:**
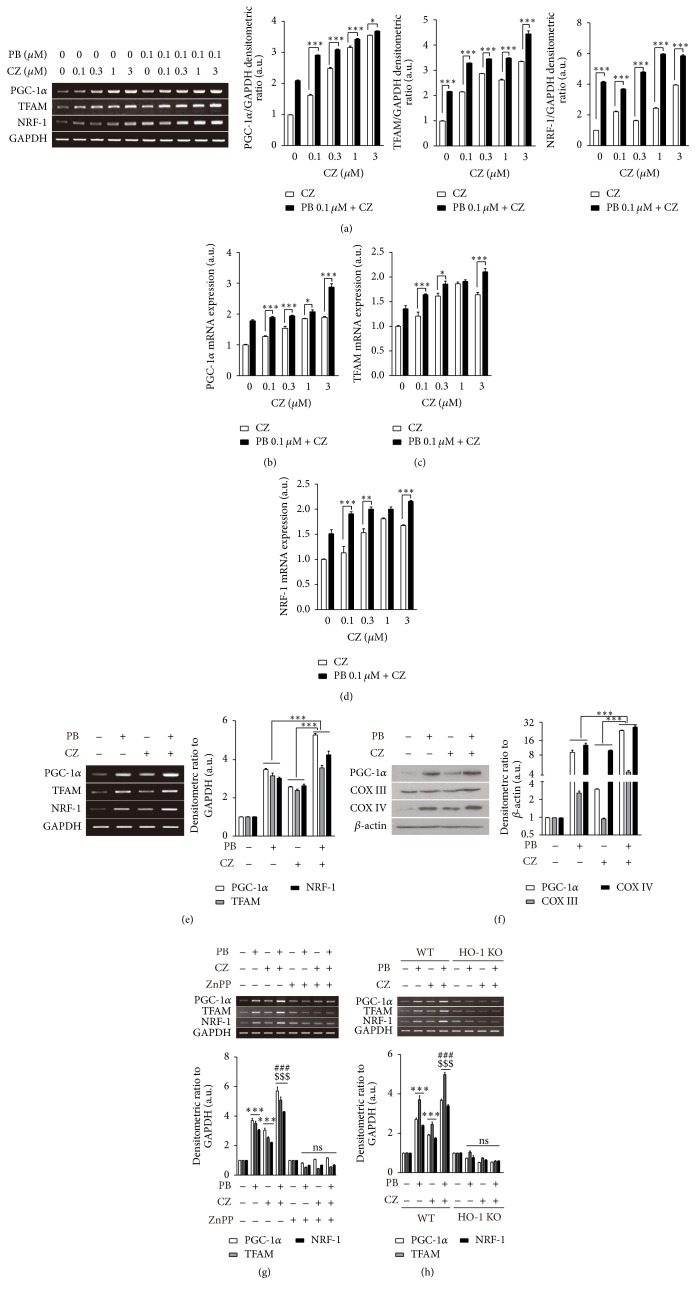
Effect of combinatorial treatment of cilostazol (CZ) and probucol (PB) on mitochondrial biogenesis-related genes expression. HepG2 cells were treated with cilostazol at various concentrations (0, 0.1, 0.3, 1, and 3 *μ*M) with or without probucol (0.1 *μ*M) for 4 h and PGC-1*α*, TFAM, and NRF-1 mRNA expression were detected by RT-PCR (a) and real-time RT-PCR (b, c, and d). Primary hepatocytes isolated from C57BL/6 mice were treated with probucol (0.1 *μ*M) and cilostazol (3 *μ*M) individually as well as with their combination for 4 h. Mitochondrial biogenesis-related genes PGC-1*α*, TFAM, and NRF-1 were measured by RT-PCR (e), and mitochondrial-related proteins PGC-1*α*, COX III, and COX IV were measured by Western blotting (f). Primary hepatocytes from HO-1 WT mice were pretreated in the absence or presence of ZnPP (10 *μ*M) and then were treated with probucol (0.1 *μ*M) and cilostazol (3 *μ*M) individually or in combination for 4 h and then the expression of mitochondrial biogenesis-related genes PGC-1*α*, TFAM, and NRF-1 was detected by RT-PCR (g). Furthermore, primary hepatocytes from HO-1 KO mice were also analyzed by RT-PCR to detect mitochondrial biogenesis-related genes expression (h). Bar graphs, right panels of (a), (e), and (f), as well as lower panels of (g) and (h) are summary data of normalized densitometric ratios. Quantitative data are expressed as means ± SE; *n* = 3. ^*∗*^ *P* < 0.05, ^*∗∗*^ *P* < 0.01, and ^*∗∗∗*^ *P* < 0.001 versus cells without treatment; ### versus cells treated with probucol; $$$ versus cells treated with cilostazol.

HO-1 regulates cardiac mitochondrial biogenesis* via *Nrf2-mediated transcriptional control of NRF-1 [9]. We have shown that cilostazol attenuates hepatic ischemia/reperfusion injury* via* HO-1 dependent activation of mitochondrial biogenesis [11]. According to previous studies, we next assessed the expression of mitochondrial biogenesis-related genes PGC-1*α*, TFAM, and NRF-1 in response to the combined treatment of cilostazol and probucol. HepG2 cells were treated with cilostazol at various concentrations (0, 0.1, 0.3, 1, and 3 *μ*M) in the absence or presence of probucol (0.1 *μ*M) for 4 h. RT-PCR (Figure 2(a)) and real-time RT-PCR (Figures 2(b), 2(c), and 2(d)) were performed to detect mRNA expression of mitochondrial biogenesis-related genes. As expected, the combined treatment of cilostazol and probucol significantly enhanced PGC-1*α*, TFAM, and NRF-1 mRNA expression compared to the corresponding dose of cilostazol alone. To confirm the beneficial effects of combinatorial treatment on mitochondrial biogenesis, we next treated primary hepatocytes with probucol (0.1 *μ*M) and cilostazol (3 *μ*M) individually as well as with their combination to test the effect on mitochondrial biogenesis-related genes at the mRNA and protein level. Consistent with results observed from HepG2 cells, combinatorial treatment of cilostazol and probucol showed a significant increase on PGC-1*α*, TFAM, and NRF-1 mRNA level (Figure 2(e)) as well as on PGC-1*α*, COX III, and COX IV protein level (Figure 2(f)) compared to their individual treatment. To evaluate whether the increase of mitochondrial biogenesis was mediated by HO-1, we pretreated primary hepatocytes with ZnPP, an inhibitor of HO activity, for 30 min. Additionally, we treated primary hepatocytes from HO-1 WT and KO mice with probucol (0.1 *μ*M) and cilostazol (3 *μ*M) for 4 h to assess the mRNA expression of PGC-1*α*, TFAM, and NRF-1. As shown in Figures 2(g) and 2(h), cilostazol and probucol had a synergistic effect on mitochondrial biogenesis-related genes, whereas they failed to increase these genes in the presence of ZnPP or in the absence of HO-1. These results indicated that cilostazol and probucol have a beneficial effect on mitochondrial biogenesis mediated by HO-1 expression.

### 3.3. Combinatorial Treatment of Cilostazol and Probucol Ameliorates Tunicamycin-Induced Mitochondrial Dysfunction

**Figure 3:**
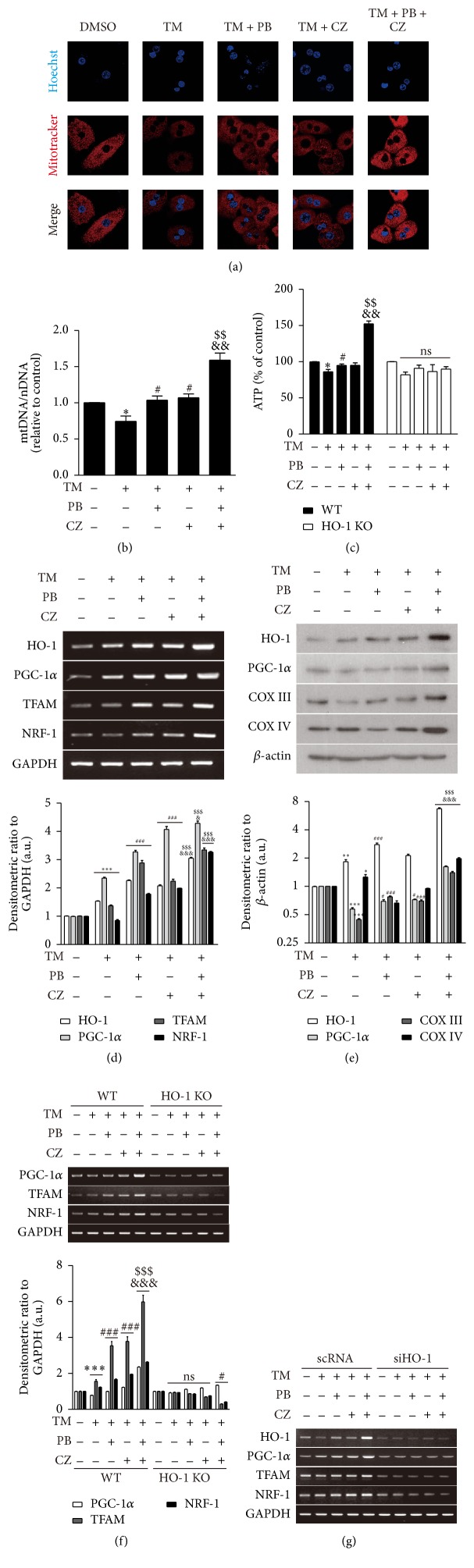
Combinatorial treatment of cilostazol (CZ) and probucol (PB) ameliorates tunicamycin- (TM-) induced mitochondria dysfunction. Primary hepatocytes were pretreated with probucol (0.1 *μ*M) and cilostazol (3 *μ*M) individually as well as with their combination for 30 min followed by stimulation of tunicamycin (10 *μ*g/mL) for 18 h. Mitochondrial mass was assessed by using MitoTracker Red (red). Nuclei were stained with Hoechst dye (blue). Images of fluorescence were analyzed by confocal microscopy (a). The relative mtDNA content was measured by real-time PCR. mtDNA content was normalized by nDNA content (b). ATP production was measured in primary hepatocytes isolated from HO-1 WT and KO mice (c). The expression of HO-1 and mitochondrial biogenesis-related genes PGC-1*α*, TFAM, and NRF-1 as well as related proteins PGC-1*α*, COX III, and COX IV was measured by RT-PCR (d) and Western blot analysis (e). RT-PCR was also performed in HO-1 KO mice to detect mitochondrial biogenesis-related genes PGC-1*α*, TFAM, and NRF-1 (f). HepG2 cells were transfected with scRNA and siHO-1 for 24 h. Then cells were pretreated with probucol (0.1 *μ*M) and cilostazol (3 *μ*M) individually or combinatorially for 30 min followed by stimulation of tunicamycin (10 *μ*g/mL) for another 18 h. RT-PCR was performed to detect HO-1, PGC-1*α*, TFAM, and NRF-1 mRNA levels (g). Bar graphs, lower panels of (d) and (e), as well as lower panel of (f), are summary data of normalized densitometry ratios. Quantitative data are expressed as means ± SE; *n* = 3. ^*∗*^ *P* < 0.05, ^*∗∗*^ *P* < 0.01, and ^*∗∗∗*^ *P* < 0.001 versus cells without treatment; ^#^ *P* < 0.05 and ^###^ *P* < 0.001 versus cells treated with tunicamycin; ^$$^ *P* < 0.01 and ^$$$^ *P* < 0.001 versus cells treated with probucol and tunicamycin; ^&^ *P* < 0.05, ^&&^ *P* < 0.01, and ^&&&^ *P* < 0.001 versus cells treated with cilostazol and tunicamycin.

Endoplasmic reticulum (ER) and mitochondria exist in physical proximity, which supports communication between these two organelles, including synthesis and transfer of lipids, exchange of calcium ions, mitochondrial ATP production, and apoptosis. Stressors on the ER can induce mitochondrial damage [26]. Therefore, we hypothesized that the combinatorial treatment of cilostazol and probucol may ameliorate ER stress-induced mitochondrial dysfunction* via* the induction of mitochondrial biogenesis. To validate the protective effect of cilostazol and probucol on mitochondrial function, we pretreated primary hepatocytes with probucol (0.1 *μ*M) and cilostazol (3 *μ*M) alone as well as with their combination for 30 min followed by stimulation with tunicamycin (10 *μ*g/mL), an ER stress inducer, for another 18 h. The quantity change of mitochondria and mtDNA content was detected in primary hepatocytes by MitoTracker staining (Figure 3(a)) and real-time PCR (Figure 3(b)), respectively. Our results showed that ER stress induced by tunicamycin reduced MitoTracker-positive staining and mtDNA content. However, the combinatorial treatment of cilostazol (3 *μ*M) and probucol (0.1 *μ*M) significantly increased mtDNA quantity and MitoTracker-positive staining compared with their individual treatments in tunicamycin-challenged cells. In addition, the combinatorial treatment of cilostazol and probucol also significantly increased ATP production during stimulation with tunicamycin, whereas it failed to enhance ATP production in the absence of HO-1 (Figure 3(c)). We further examined the expression of HO-1 and mitochondrial biogenesis-related genes PGC-1*α*, TFAM, and NRF-1 as well as related proteins PGC-1*α*, COX III, and COX IV by RT-PCR (Figure 3(d)) and Western blot (Figure 3(e)), respectively. Combinatorial treatment of cilostazol and probucol in primary hepatocytes significantly increased these genes and proteins during tunicamycin challenge compared with their individual treatments alone. In contrast, these gene expression changes were not evident in HO-1 deficient cells (Figure 3(f)) or in HepG2 cells transfected with HO-1 siRNA (Figure 3(g)). These results suggest that cilostazol and probucol have a synergistic protective effect on ER stress-induced mitochondrial dysfunction* via* promotion of mitochondrial DNA biogenesis, ATP production, and related gene expression, which was mediated by HO-1 expression.

### 3.4. Combinatorial Treatment of Cilostazol and Probucol Ameliorates Tunicamycin-Induced Hepatosteatosis in Mouse Model

**Figure 4:**
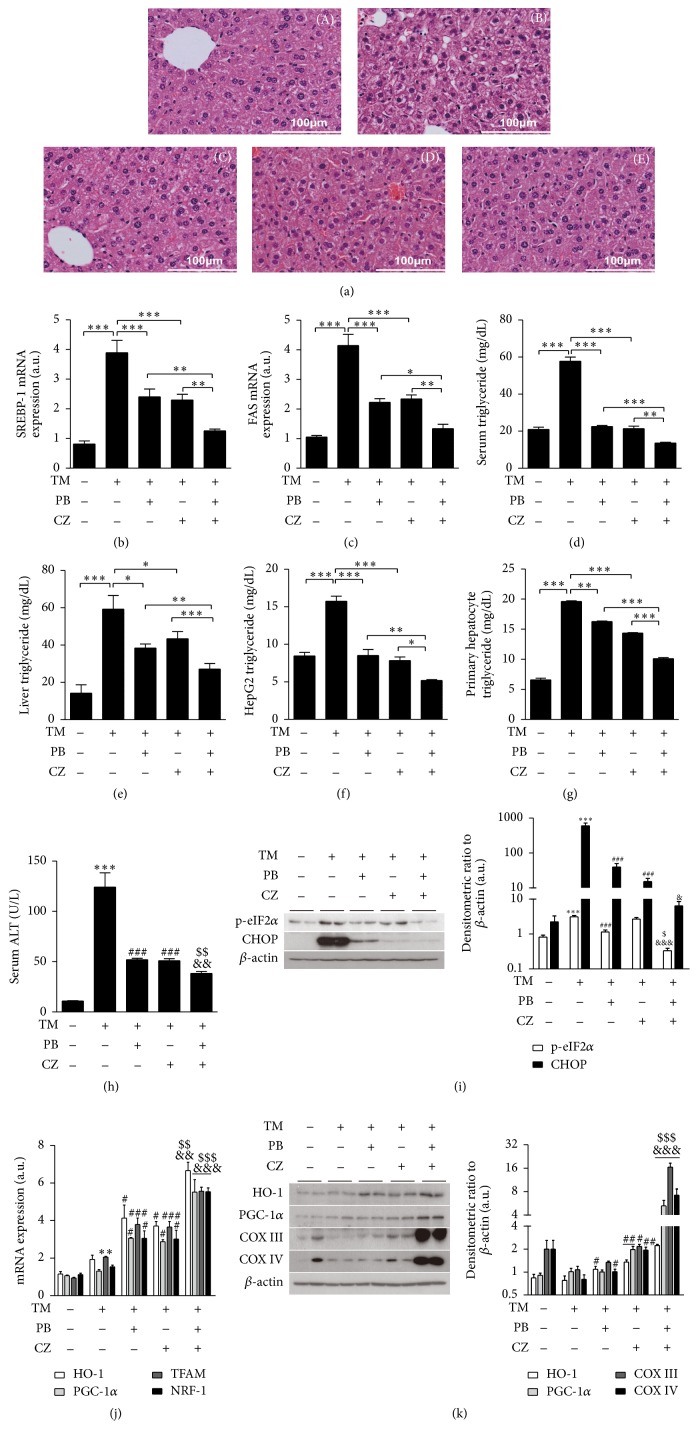
Combinatorial treatment of cilostazol (CZ) and probucol (PB) ameliorates tunicamycin- (TM-) induced hepatosteatosis* in vivo*. C57BL/6 mice were pretreated with cilostazol (3 mg/kg body weight) and probucol (1 mg/kg body weight) individually or combinatorially once daily for 3 days by intraperitoneal injection. The mice were sacrificed after challenge with tunicamycin (3 mg/kg body weight) for 24 h. Liver tissues were excised and representative liver histology is shown by H&E staining (a). Liver sections were, respectively, from CON (A), TM (B), TM + PB (C), TM + CZ (D), and TM + PB + CZ (E) treated mice. The expression of lipogenesis-related genes SREBP-1c and FAS in liver was measured by real-time RT-PCR (b and c). Triglyceride levels of serum (d), liver tissues (e), HepG2 cells (f) and primary hepatocytes (g), and ALT concentrations (h) were measured. For liver ER stress related proteins, p-eIF2*α* and CHOP were measured by Western blot (i). The expression of HO-1 and mitochondrial biogenesis-related genes PGC-1*α*, TFAM, and NRF-1, as well as related proteins PGC-1*α*, COX III, and COX IV, was measured by real-time RT-PCR (j) and Western blot (k), respectively. Bar graphs, left panels of (i) and (k), are summary data of normalized densitometric ratios. All mice were separated into experimental groups (*n* = 6 mice per group). Quantitative data are expressed as means ± SE; *n* = 3. ^*∗*^ *P* < 0.05, ^*∗∗*^ *P* < 0.01, and ^*∗∗∗*^ *P* < 0.001 versus cells without treatment; ^#^ *P* < 0.05, ^##^ *P* < 0.01, and ^###^ *P* < 0.001 versus cells treated with tunicamycin; ^$^ *P* < 0.05, ^$$^ *P* < 0.01, and ^$$$^ *P* < 0.001 versus cells treated with probucol and tunicamycin; ^&^ *P* < 0.05, ^&&^ *P* < 0.01, and ^&&&^ *P* < 0.001 versus cells treated with cilostazol and tunicamycin.

Increasing evidence suggests that hepatic ER stress increases nonalcoholic fatty liver disease (NFALD) in several animal models. Moreover, ER stress leads to lipid accumulation through upregulation of lipogenesis-related genes SREBP-1c and FAS in normal hepatic and hepatoma cells [27, 28]. To confirm the combinatorial effect of cilostazol and probucol on ER stress-induced hepatic steatosis, we injected C57BL/6 mice with cilostazol (3 mg/kg body weight) and probucol (1 mg/kg body weight) individually or combinatorially once daily for 3 days then sacrificed mice after challenging them with tunicamycin (3 mg/kg body weight) for 24 h. In agreement with the histological analysis, lipid accumulation and liver injury were evident in tunicamycin-treated mice. However, the combinatorial treatment of cilostazol and probucol markedly decreased lipid accumulation and improved the ballooning degeneration of hepatocytes in response to tunicamycin stimulation when compared to their individual treatments (Figure 4(a)). Furthermore, we tested SREBP-1c (Figure 4(b)) and FAS (Figure 4(c)) mRNA expression in liver tissues as well as serum triglyceride concentration (Figure 4(d)), liver tissues (Figure 4(e)), HepG2 cells (Figure 4(f)), and primary hepatocytes (Figure 4(g)). The increased mRNA level of SREBP-1c and FAS and serum triglyceride concentration were significantly decreased by combinatorial treatment of cilostazol and probucol compared with their individual treatments. To evaluate the liver damage induced by tunicamycin, we next detected serum ALT concentration (Figure 4(h)) and protein level of the ER stress markers p-eIF2*α* and CHOP (Figure 4(i)) in liver tissues. Consistent with the observed results, combination of cilostazol and probucol drastically decreased the serum ALT concentration, as well as p-eIF2*α* and CHOP expression under the challenge of tunicamycin. Moreover, the expression of HO-1 and mitochondrial biogenesis-related genes was detected by real-time RT-PCR (Figure 4(j)) and Western blot analysis (Figure 4(k)). Combinatorial treatment of cilostazol and probucol significantly increased HO-1 and mitochondrial biogenesis-related genes under the tunicamycin stimulation in liver tissues compared to their individual treatments. Taken together, these results suggested that cilostazol and probucol exhibited synergistic effect on protection of hepatic steatosis caused by the ER stress inducer tunicamycin.

## 4. Discussion

Mitochondrial biogenesis is defined as the growth and division of preexisting mitochondria, which can be induced in adult muscle in response to exercise or chronic electrical stimulation. This kind of physiological control is considered as an adaptation to facilitate increased oxygen utilization [29, 30]. Reduction in mitochondrial function has been found to be associated with the pathology of several human diseases, such as type 2 diabetes and Alzheimer's disease [31]. Also, our laboratory found that mitochondrial dysfunction was associated with hepatic ischemia/reperfusion injury [11]. Mitochondria play an important role in hepatocyte metabolism, being the primary site for the oxidation of fatty acids and oxidative phosphorylation. Accumulation of FFAs in the liver is implicated in the pathogenesis of NAFLD [6]. Although the mechanisms for the pathogenesis of NAFLD are still unknown, they may be related to mitochondrial dysfunction including depletion of mitochondrial DNA (mtDNA), decreased activity of respiratory chain complexes, and impaired mitochondrial *β*-oxidation [32–34]. Moreover, NAFLD is caused by ER stress-induced mitochondrial dysfunction [35, 36]. To preserve the functional integrity of mitochondria, HO-1 plays an important role in recovering impaired mitochondria by ER stress [27].

Heme oxygenase (HO) is the rate-limiting enzyme in the degradation of heme, which generates biliverdin, carbon monoxide (CO), and iron. Two distinct isoforms of HO have been identified and cloned including HO-1 and HO-2 [28]. Previous studies have shown the critical importance of HO-1 expression in mediating antioxidant, anti-inflammatory, and antiapoptotic effects [37–39]. Recent studies have demonstrated the coupling of mitochondrial biogenesis to anti-inflammation through HO-1 activity in a murine sepsis model [40]. HO-1 has been also suggested to regulate mitochondrial biogenesis* via* NF-E2-related factor-2- (Nrf2-) mediated transcriptional control of nuclear respiratory factor-1 (NRF-1) as well as the PGC-1*α* coactivator and mitochondrial transcription factor A (TFAM) in cardiomyocytes [9, 41]. For this reason, we have studied the synergistic effect of cilostazol and probucol on the HO-1 expression.

Cilostazol, as a selective inhibitor of PDE3 used to increase the intracellular level of cAMP [12], is widely used for the treatment of thrombotic vascular disease due to its antiplatelet aggregation functions [13]. In recent studies, cilostazol was shown to induce HO-1 expression to inhibit inflammation in J774 murine macrophages* via *the Nrf2 and PI3K/AKT signal pathways [42]. Probucol is a potent lipid-soluble antioxidant, which has been reported to possess strong antiatherogenic properties [18]. Also probucol increases HO-1 expression and activity in balloon-injured rabbit aortas and rabbit aortic smooth muscle cells, which confers protection from atherosclerosis [22] and inhibits smooth muscle cell proliferation [21]. Although previous studies have demonstrated that either cilostazol or probucol upregulates HO-1 expression, little is known on the combinatorial effect of these two chemicals on HO-1 production as well as on mitochondrial DNA biogenesis. In this study, HepG2 cells were initially treated with a combination of cilostazol and probucol, and we found that the combinatorial effect on increasing HO-1 mRNA expression is much higher than the effect of cilostazol alone especially at the optimal combination doses (3 *μ*M and 0.1 *μ*M), respectively.

To investigate the synergistic effect of cilostazol and probucol in activating mitochondrial biogenesis* via* induction of HO-1, the expression of PGC-1*α*, TFAM, NRF-1, complex III, and complex IV was measured in HepG2 cells or primary hepatocytes from wild-type or HO-1 knockout mice (*Hmox1* ^−/−^) (Figure 2). Mitochondrial biogenesis, which is controlled by several key factors including PGC-1*α*, maintains mitochondrial populations and mitochondrial homeostasis. PGC-1*α* acts as a cardinal transcriptional regulator of mitochondrial biogenesis by activating nuclear respiratory factor-1 (NRF-1). TFAM activated by PGC-1*α* and NRF-1 regulates the transcription of nuclear genes encoding mitochondrial proteins [43, 44]. The mitochondrial abnormalities in liver also are related to the low levels of complexes I, III, IV, and V [8]. By measuring the levels of PGC-1*α*, TFAM, NRF-1, complex III, and complex IV in the deficiency of HO-1, we have found that HO-1 plays a critical role in the synergic effects of cilostazol and probucol on mitochondrial biogenesis.

ER stress promotes lipid drop formation in human adipose and liver tissue [6] and may promote NAFLD and metabolic syndrome [45]. Tunicamycin, as an ER stressor, decreases mitochondrial biogenesis (Figure 3) and increases lipogenesis* in vivo *(Figure 4). ER stress-induced ROS [46] may induce HO-1 expression as defense against oxidative stress in the early stage [47] and also increase PGC-1*α* and PGC-1*β* for regulation of ROS defense mechanisms [48]. Therefore, although HO-1 and PGC-1 expressions were induced by tunicamycin to some degree in our experiments, this was not sufficient to protect mitochondria against functional impairment by ER stress. Finally, combinatorial treatment of cilostazol and probucol improved mitochondrial dysfunction* via* enhanced HO-1 expression in the presence of ER stress.

Taken together, we demonstrate for the first time that cilostazol and probucol have a beneficial synergistic effect on HO-1 mRNA expression and on activation of mitochondrial biogenesis at relatively low doses. These findings provide new clues for the development of new therapeutics aimed at metabolic diseases.
